# Anticancer and Anti-Metastatic Role of Thymoquinone: Regulation of Oncogenic Signaling Cascades by Thymoquinone

**DOI:** 10.3390/ijms23116311

**Published:** 2022-06-05

**Authors:** Ammad Ahmad Farooqi, Rukset Attar, Baojun Xu

**Affiliations:** 1 Department of Molecular Oncology, Institute of Biomedical and Genetic Engineering, Islamabad 54000, Pakistan; farooqiammadahmad@gmail.com; 2 Department of Obstetrics and Gynecology, Yeditepe University, Istanbul 34280, Turkey; ruksetattar@hotmail.com; 3 Food Science and Technology Program, BNU-HKBU United International College, Zhuhai 519087, China

**Keywords:** anticancer, thymoquinone, anti-metastasis, signaling networks

## Abstract

Cancer is a life-threatening and multifaceted disease. Pioneering research works in the past three decades have mechanistically disentangled intertwined signaling networks which play contributory roles in carcinogenesis and metastasis. Phenomenal strides have been made in leveraging our scientific knowledge altogether to a new level of maturity. Rapidly accumulating wealth of information has underlined a myriad of transduction cascades which can be pharmaceutically exploited for cancer prevention/inhibition. Natural products serve as a treasure trove and compel interdisciplinary researchers to study the cancer chemopreventive roles of wide-ranging natural products in cell culture and preclinical studies. Experimental research related to thymoquinone has gradually gained momentum because of the extra-ordinary cancer chemopreventive multifunctionalities of thymoquinone. In this mini-review, we provide an overview of different cell signaling cascades reported to be regulated by thymoquinone for cancer chemoprevention. Essentially, thymoquinone efficacy has also been notably studied in animal models, which advocates for a rationale-based transition of thymoquinone from the pre-clinical pipeline to clinical trials.

## 1. Introduction

Deregulated cell signaling pathways play fundamental roles in re-shaping multiple facets of cancer, encompassing a wide range of functions that include the early initiation stage through to the metastatic stage, drug resistance, loss of apoptosis, and genetic/epigenetic inactivation [1,2,3,4]. Ground-breaking discoveries associated with the extra-ordinary pharmaceutical and medicinal values of natural products have catalyzed outstanding breakthroughs in drug development, consequently resulting in the rapid maturation of generalizable chemical platforms for the multi-targeting of previously undruggable oncogenic proteins [5,6,7,8,9]. 

Thymoquinone is isolated from *Nigella sativa* with a molecular weight of 164.2 g/mol. A series of cutting-edge research works have demonstrated the health-promoting and disease-ameliorating roles of thymoquinone [10,11,12,13,14]. Although different research teams have reviewed cancer chemopreventive role of thymoquinone [15,16,17,18,19], we summarize mechanistic insights gained through cell culture studies and xenografted mice. Herein, we provide a comprehensive overview of the fast-evolving field of pharmacological targeting of signaling pathways by thymoquinone in cancer and discuss the possibilities associated with targeting JAK/STAT, Wnt/β-catenin, PI3K/AKT/mTOR, NF-κB, and TRAIL-driven pathways for drug development in a wide range of oncology settings. We underline the knowledge gaps, identify key challenges, and make recommendations on how to rationally accelerate laboratory findings into clinically effective therapeutics.

For the framework of the mini-review, we extensively browsed PubMed using diverse keywords, particularly, “thymoquinone and cancer”, “thymoquinone and metastasis”, and “thymoquinone and signaling”. 

In the upcoming sections, we will summarize thymoquinone-mediated regulation of cell signaling pathways in different cancers. 

## 2. Regulation of JAK/STAT

A multimodule gene regulatory network mediates the complex process, involving signaling events and regulation of target genes, which modulates carcinogenesis and metastasis. The JAK/STAT pathway has been reported to be extensively involved in carcinogenesis and the spread of cancer cells to distant organs [20,21,22]. This section mainly deals with the thymoquinone-mediated inhibitory effects on the JAK/STAT pathway. 

Thymoquinone inhibited the JAK2-mediated phosphorylation of STAT3 on the 727th serine residue in SK-MEL-28 cells. Importantly, levels of cyclin D1, D2, and D3 were reported to be reduced in STAT3-depleted SK-MEL-28 cells. Intraperitoneally administered thymoquinone caused tumor shrinkage in mice inoculated with SK-MEL-28 cells [23]. 

Gamma knife has been shown to be an effective treatment against brain metastasis from melanoma. Brain metastasis commonly occurs in patients suffering from metastatic malignant melanoma. Essentially, conversion of transient remissions to stable cures remains an overarching goal for clinical investigators of melanoma. Gamma knife and thymoquinone combinatorially enhanced the survival rates of C57BL/6J mice with intra-cerebral B16-F10 melanoma [24].

Furthermore, thymoquinone has also been reported to increase the survival rates in C57BL/6J mice with intracerebral B16-F10 melanoma cells. The JAK2/STAT3 pathway is inactivated by thymoquinone in B16-F10 melanoma cells. Collectively, these results clearly indicate that pharmacological targeting of melanoma brain metastasis by thymoquinone needs to be tested more comprehensively [25]. 

**Figure 1:**
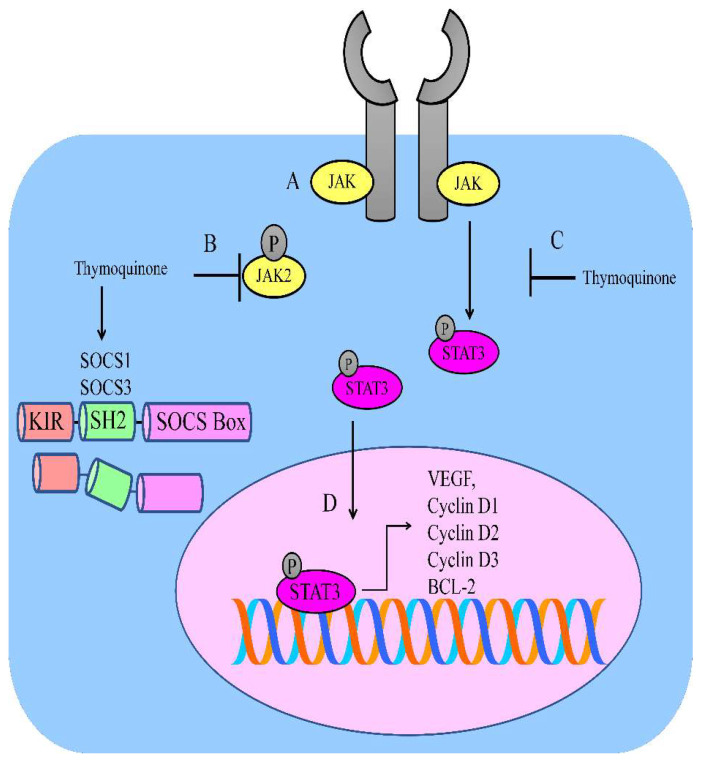
(A) JAK/STAT-mediated signaling is regulated by thymoquinone. (B,C) Thymoquinone inhibited JAK2 and STAT3. Thymoquinone stimulated the expression of SOCS1 and SOCS3. (D) STAT3-mediated upregulation of VEGF; BCL-2; and cyclin D1, D2, and D3.

Renal cell carcinoma cells have high levels of anti-apoptotic proteins (Chae). It has also been noted that thymoquinone blocked the JAK2/STAT3-mediated upregulation of BCL-2, survivin, and cyclin D2 in Caki-1 cells (Figure 1). Intraperitoneal injections of thymoquinone hampered the growth of Caki-1 cell xenografts in nude mice [26]. 

Similarly, intraperitoneally administered thymoquinone impaired tumor growth rates in NSG mice xenotransplanted with epidermoid carcinoma A431 cells. Importantly, p53 is negatively regulated by MDM2. It was noted that thymoquinone increased p53 levels by simultaneous suppression in the level of MDM2 [27]. 

Thymoquinone inactivated the JAK2/STAT3 pathway in gastric HGC27 cancer cells. Thymoquinone also suppressed STAT3 target genes, such as survivin, VEGF, cyclin D, and BCL-2 (Figure 1). Intraperitoneally administered injections of thymoquinone induced tumor shrinkage in xenografted models [28]. 

Thymoquinone and cisplatin induced the regression of tumor xenografts. Importantly, p-STAT3 levels were noted to be profoundly reduced in the tumor tissues of thymoquinone and cisplatin-treated mice [29]. 

Evidence suggests that thymoquinone is efficient against acute myeloid leukemia. Thymoquinone chemically interacted with the active pockets of JAK2, STAT3, and STAT5 and inhibited their activities. Over the past two decades, following the discovery of the SOCS protein family, a wealth of knowledge has uncovered the functions and structures of SOCS proteins. SOCS proteins are negative-feedback regulators and a thymoquinone-mediated increase in SOCS proteins is necessary for inactivation of STAT proteins. Thymoquinone stimulated the expression of SHP-1, SOCS1, and SOCS3 in MV4–11 cells (Figure 1) [30]. 

## 3. Wnt/β-Catenin Signaling

With the advancements in sequencing technologies and detailed structural characterization of the cancer genome, it is apparent that WNT pathway mutations frequently occur in various cancers. β-Catenin is widely acclaimed as the principal transducer of canonical WNT signals to the nucleus. The orchestrated co-operation between cell surface mechanics and intracellular signaling has significant impacts on biological processes. In the absence of WNT ligands, β-catenin is phosphorylated by GSK3β, ubiquitylated by β-TrCP, and marked for degradation. The classical pathway is switched “on” upon binding of WNT ligands to frizzled receptors and LRP co-receptors, which results in stabilization and accumulation of β-catenin [31,32,33,34]. 

Thymoquinone dose-dependently inhibited nuclear accumulation of β-catenin [35]. Levels of β-catenin and Wnt/β-catenin target genes, such as c-Myc, matrix metalloproteinase-7, and Met, were found to be reduced in thymoquinone-treated bladder cancer cells. Importantly, β-catenin overexpression drastically abrogated the repressive effects of thymoquinone on the epithelial-to-mesenchymal transition by enhancing the levels of *N*-cadherin and vimentin. Intraperitoneally injected thymoquinone caused regression of tumor mass in xenografted mice. Moreover, pulmonary metastases models were generated by injections of T24-L-tagged luciferase for evaluation of the repressive effects of thymoquinone on distant metastasis. Moreover, bioluminescence imaging clearly indicated that pulmonary metastases were inhibited considerably by thymoquinone. Thymoquinone has been shown to significantly inhibit the foci of lung metastasis [35]. 

The MITF promoter present in close vicinity to the common downstream exon is known as the M promoter and expressed selectively in melanocytes [36]. Proteasomal degradation of β-catenin occurs through GSK3β-mediated phosphorylation at the serine-33, serine-37, serine-45, and threonine-41 residues of β-catenin. Studies have shown that phosphorylation at the Tyr-216 residue of GSK3β significantly enhances the enzymatic activity of GSK3β, whereas the phosphorylation at the 9th serine residue of GSK3β inactivates it. β-catenin has previously been reported to increase MITF expression. Therefore, strategic inactivation of β-catenin resulted in a decline in MITF levels. Thymoquinone dose-dependently reduced the expression of MITF and tyrosinase that was accompanied by decreased tyrosinase activity in B16F10 cells. Pre-treatment with LiCl resulted in an increase in the levels of p-GSK3β that led to the blockade of β-catenin degradation and increased the expression and activity of tyrosinase [36].

The generation of hybrids between conventional chemotherapeutic and bioactive natural products is an innovative approach to obtain effective anticancer compounds [37]. A hybrid of thymoquinone and 5-fluorouracil not only reduced β-catenin levels but also suppressed transcriptional activity of β-catenin in colorectal cancer cell lines. HCT116 control cells developed highly vascularized tumor masses, whereas hybrid-treated-tumor xenografts were significantly smaller in size. Interesting, there were signs of cellular proliferation in the xenografts combinatorically treated with thymoquinone and 5-fluorouracil, whereas hybrid-treated tumors did not show any sign of mitotic activity [37]. 

Intraperitoneally injected thymoquinone reduced the size and number of aberrant crypt foci and tumor multiplicities in a chemical-induced model of colorectal cancer [38]. Thymoquinone reduced polyp growth and selectively induced apoptosis. High doses of thymoquinone led to the translocation of β-catenin to the membrane and a reduction in the large polyps of APC^Min^ mice [38]. 

It has been shown that the phosphorylation of GSK3β at serine-9 induced inactivation. Therefore, thymoquinone reduced p-GSK3β, β-catenin, and MMP2/MMP9 in Eca109 cells [39]. 

## 4. Regulation of UHRF1 by Thymoquinone

The ubiquitin-like protein containing PHD and RING fingers domains-1 (UHRF1) is a multi-domain-containing protein. UHRF1 maintains DNA methylation through the recruitment of DNA methyltransferase-1 to the replication forks in the S-phases of the cells. 

Thymoquinone induced an auto-ubiquitination of UHRF1 through its RING domain [40]. The protein p73 is a functional and structural homologue of p53. P73 induced apoptosis in cancer cells. However, UHRF1 epigenetically inactivated p73 in Jurkat cells. However, thymoquinone-mediated auto-ubiquitination of UHRF1 caused a sharp increase in the expression of p73 in Jurkat cells [40].

UHRF1 binds to the inverted CCAAT domain in the promoter region of TXNIP and inhibits its expression via CpG methylation [41]. UHRF1 knockdown inhibited UHRF1 binding to the promoter of TXNIP and enhanced TXNIP expression through promoter demethylation in HeLa cells. Levels of ubiquitin-specific protease-7 (USP7) were noted to be enhanced in HPV16 E6/E7-overexpressing cells. USP7 efficiently stabilized UHRF1 and epigenetically inactivated TXNIP [41]. 

Likewise, HPV E6/E7 caused a marked increase in the levels of USP7 and UHRF1. UHRF1 effectively inactivated gelsolin in HeLa cells, whereas thymoquinone induced the expression of gelsolin and promoted apoptotic death in cancer cells [42]. 

## 5. PI3K/AKT/mTOR

The PI3K/AKT/mTOR pathway has long been an attractive target in molecular oncology. Research teams have focused on the pharmacological targeting of key components of this signaling network [43,44,45,46,47]. 

Thymoquinone dose-dependently reduced the levels of p-AKT (threonine-308), p-AKT (serine-473), p-mTOR1, and p-mTOR2 in gastric cancer cells. Thymoquinone inhibited the colony formation and invasive capacities of gastric cancer cells [48]. 

Thymoquinone alone and with pre-treatment significantly reduced p-AKT in BxPC-3, AsPC-1, and PANC-1 cells [49]. Thymoquinone pre-treatment markedly impaired gemcitabine-mediated increases in the phosphorylation of mTOR and S6 in pancreatic cancer cells. More importantly, thymoquinone and gemcitabine induced shrinkage of the primary tumors in the orthotopic cancer models of PANC-1 cells. Pre-treatment with thymoquinone led to marked suppression in p-AKT, p-mTOR, and p-S6 in tumor tissues of xenografted mice [49].

AMPK activates autophagy by inhibition of mTOR [50]. The inhibition of mTORC1 increased autophagy, whereas mTORC1 activation caused deactivation of autophagy. Thymoquinone caused an increase in the level of p-AMPK in 786-O and ACHN cells, while levels of p-mTOR and p-S6K were reduced. There was a significant decline in the number of metastatic nodules in the lungs of thymoquinone-treated mice [50]. 

Thymoquinone effectively inhibited the PI3K/AKT/mTOR pathway independently and in combination with 5-fluorouracil and active vitamin D3 in colorectal cancer cells [51]. 

## 6. NF-κb Activity in Carcinogenesis: Paradoxical Roles of Thymoquinone

Learning more about the complicated mechanisms of NF-κB regulation can be advantageous in the design and development of better therapeutic approaches to target versatile transcriptional factor in different types of cancers. NF-κB functions are controlled tightly by several regulatory proteins, and a disruption of this process has been associated with carcinogenesis and metastasis [52,53,54,55]. NF-κB provides a mechanistic linkage between cancer and inflammation and is a main regulator controlling the capability of malignant cells to trigger pro-survival signaling and resist apoptotic cell death. 

Thymoquinone stimulated the expression levels of miR-603 in MDA-MB-436 and MDA-MB-231 cancer cells. NF-κB transcriptionally repressed miR-603. Importantly, miR-603 directly targeted eEF-2K and inhibited the proliferation and invasive capacities of breast cancer cells. Intravenously administered thymoquinone-loaded liposomal nanoparticles impaired tumor growth in mice orthotopically implanted with MDA-MB-231 and MDA-MB-436 cancer cells [56]. 

Thymoquinone and bortezomib combinatorially induced tumor shrinkage in mice subcutaneously implanted with U266 cells. NF-κB activity was significantly reduced in the tumor tissues of mice xenografted with multiple myeloma cells [57]. 

There was a significant decrease in the ratio of p-NF-κB/NF-κB in tumor xenografts from mice combinatorially treated with thymoquinone and cisplatin [58] 

Thymoquinone inhibited NF-κB-mediated activity and prevented cancer progression [59,60]. 

*Cancer-promoting role of Thymoquinone:* Prolonged thymoquinone treatment results in an increase in NF-κB reporter activities and induced a two-fold rise in volume of the ascites [61]. 

ID8-NGL mouse ovarian cancer cells stably expressing NF-κB have been investigated to analyze the cancer chemopreventive effects of thymoquinone. ID8-NGL cells were intraperitoneally injected into a C57BL/6 rodent model. Prolonged treatment of thymoquinone (30 days) resulted in an increase in the activity of NF-κB in tumors. Moreover, prolonged treatment of thymoquinone induced pro-tumor M2-like macrophages within the tumor microenvironments. Thymoquinone triggered an increase in the infiltration of macrophages. Thymoquinone caused an overall increase in the expression of IL-1β and TNFα in macrophages and VEGF in ascites fluid. M2-like macrophages produced high concentrations of signaling molecules such as TNFα, which increased the activity of NF-κB in the tumor tissues, consequently resulting in drug resistance in a sub-population of cancer cells [62]. 

## 7. Chemokine Ligand-Driven Signaling

**Figure 2:**
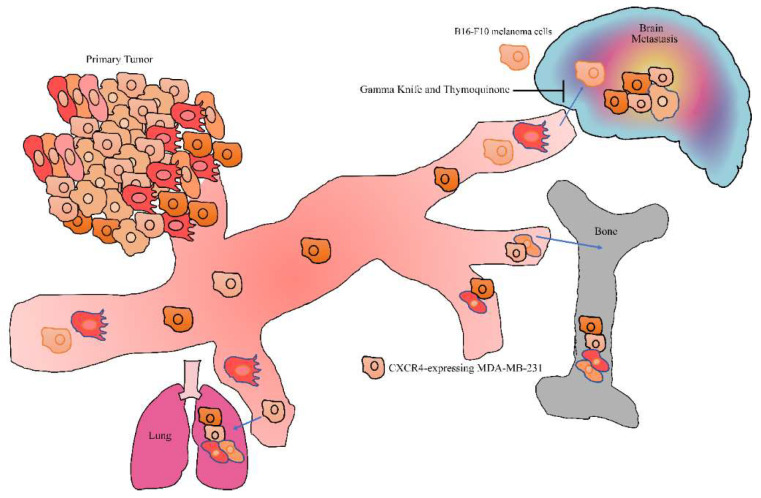
Metastatic spread of cancer cells to distant organs.

Thymoquinone effectively inhibited NF-κB-mediated transcriptional upregulation of CXCR4 [63]. Importantly, thymoquinone caused a marked reduction in the number of colonies metastasized to secondary sites. Moreover, CXCR4-expressing MDA-MB-231 cancer cells showed a tendency to migrate towards a CXCL12-expressing microenvironment in mice intracardially implanted with MDA-MB-231 cells. Thymoquinone treatment led to significant suppression of the metastatic colonization of breast cancer cells into the bones (Figure 2). Moreover, there was an evident reduction in metastatic sites, such as the bone marrow of the tibiae and femora, as well as the mandibles. Similarly, thymoquinone-mediated anti-metastatic effects were also observed in the lungs and brain tissues [63].

CXCL12 induced a physical association between CXCR4 and CD45 in multiple myeloma cells [64]. Multiple CD45-silenced myeloma cells completely lost their migratory potential in response to CXCL12. Thymoquinone decreased the surface expression of CXCR4 on multiple myeloma cells and CXCL12-mediated CXCR4-CD45 interactions [64]. 

## 8. Regulation of TRAIL-Mediated Signaling by Thymoquinone

The tumor necrosis factor-related apoptosis ligand (TRAIL) triggered apoptotic death through engagement of the death receptors DR4 and DR5 [65,66,67,68,69,70].

Thymoquinone effectively enhanced TRAIL-mediated DNA damage in HepG2 cells. Thymoquinone and TRAIL synergistically reduced the levels of XIAP (X-linked inhibitor of apoptosis), c-FLIP, BCL-2, cIAP1, and cIAP2 in HepG2 cancer cells [71]. Different studies have shown that Thymoquinone stimulates the expression of death receptors for activation of intracellular apoptotic signaling in HepG2 cells. Moreover, there is sufficient evidence for TRAIL-mediated activation of NF-κB in different cancers. However, thymoquinone efficiently reduced the levels of NF-κB [72,73].

Activated B-cell lymphoma (ABC), a subtype of diffuse large B-cell lymphoma (DLBCL), has a poor survival rate. Thymoquinone markedly reduced serine-32-phosphorylated IκBα. The phosphorylation of IκBα led to the release of NF-κB and the consequent activation of NF-κB-target genes [74].

Thymoquinone time-dependently decreased BCL-2 levels and simultaneously enhanced BAX levels [75]. Thymoquinone promoted the release of mitochondrially located cytochrome c to the cytosol in primary effusion lymphoma (PEL) cell lines (BC1 and BC3). N-acetyl cysteine caused a blockade of conformational changes in BAX protein. Essentially, thymoquinone induced structural re-orientation necessary for the activation of BAX. Thymoquinone-mediated ROS accumulation triggered conformational changes in BAX that sequentially resulted in the activation of the mitochondrial apoptotic pathway. Thymoquinone effectively increased the release of cytochrome c into the cytosol [75].

## 9. Regulation of EMT by Thymoquinone

The epithelial-to-mesenchymal transition (EMT)-associated reprogramming of cells not only showcases fundamental changes in different regulatory networks but also informs us about an intricate interplay that exists between them. Deregulation of a controlled epithelial balance is triggered by alterations in several regulatory layers. Our rapidly evolving conceptualization of the genetic evolution of metastatic diseases has enabled us to decode variability in the therapeutic vulnerabilities of primary and secondary/metastatic tumors [76,77,78]. Excitingly, cutting-edge and seminal researches have shed light on the routes to and temporal patterns of metastatic colonization and produced mechanistic insights into the complex barcodes which underlie metastasis [79,80].

Myrtucommulone-A and thymoquinone notably reduced the levels of vimentin, Slug, and N-cadherin, whereas levels of E-cadherin were found to be enhanced [81]. Thymoquinone downregulated TWIST1 and ZEB1 and simultaneously upregulated E-cadherin in SiHa and CaSki cell lines [82]. 

## 10. Regulation of the MAPK Pathway by Thymoquinone:

The MAPK transduction pathway encompasses a cascade of phosphorylation events involving three functionally active key kinases, namely RAF, MEK, and ERK [83,84,85,86,87,88,89,90]. Importantly, this exciting kinase cascade presents unique prospects for the design and development of novel and effective cancer therapeutics. 

Thymoquinone induced an increase in the phosphorylated levels of JNK, p38-MAPK, and ERK. Thymoquinone-induced ROS enhanced the phosphorylation of p38-MAPK in MCF-7 cells. Thymoquinone-mediated apoptosis was impaired in p38-MAPK silenced cancer cells [91]. 

It had previously been shown that the transfection of cells with the non-phosphorylatable mutants T212A caused an increase in p-PAK(threonine-423) and enhanced apoptotic death [92]. Similarly, an increase in apoptosis was noted in cells transfected with kinase-dead K299R mutants and PAK1 siRNAs. The thymoquinone-induced activation of ERK1/2 is inhibited when PAK (threonine-423) is phosphorylated maximally. Secondly, the combination of IPA-3 (PAK inhibitor) with thymoquinone led to a decrease in the phosphorylation of threonine-423, accompanied by a significant rise in p-ERK1/2 levels that highlighted an early activation of the MEK-ERK cascade. Moreover, the lack of inhibition of p-PAK1 (threonine-423) is associated closely with a decrease in pro-survival ERK1/2 activation and a considerable increase in apoptotic cell death [92]. 

FR180204 (ERK inhibitor) significantly reduced the viability of thymoquinone and docetaxel-treated cancer cells [93]. Collectively, these findings indicate that ERK inhibition/inactivation reduced the viability of cancer cells. 

Thymoquinone inhibited the proliferation, migration, and invasion of A549 cells by inactivating the ERK1/2 signaling cascade [94]. ERK and JNK activation protected DLD-1 cells from thymoquinone-induced oxidative stress and apoptotic cell death [95]. Therefore, a combination of thymoquinone with ERK and JNK inhibitors can efficiently enhance the cancer-inhibitory effects of thymoquinone. 

## 11. Regulation of microRNAs by Thymoquinone

The discovery of microRNAs has altered our perception of non-coding RNAs from ‘junk’ transcriptional products to functional regulatory molecules [96,97,98]. 

PD-L1 is directly targeted by hsa-miR-877-5p. Thymoquinone promoted hsa-miR-877-5p-driven targeting of PD-L1 in T24 and 5637 bladder cancer cells. miR-877-5p inhibitors enhanced the migratory capacity of bladder cancer cells and weakened thymoquinone-induced inhibitory effects on the invasive potential of cancer cells. Thymoquinone and cisplatin induced tumor retrogression in mice inoculated with T24 cancer cells. hsa-miR-877-5p levels were found to be enhanced in tumor xenografts in mice inoculated with T24 cells. Moreover, thymoquinone profoundly suppressed the number of pulmonary metastatic nodules in mice injected with bladder cancer cells [99]. 

Src family kinases are nonreceptor tyrosine kinases involved in the modulation of many signaling cascades. Thymoquinone markedly reduced the levels of eEF-2K and suppressed the phosphorylated levels of Src and FAK [56]. Knockdown of eEF-2K caused significant inhibition of the migratory and invasive potential of MDA-MB-436 and MDA-MB-231 cancer cells. Importantly, there was a significant reduction in the levels of p-Src, p-AKT, p-FAK, and p-EF2 in MDA-MB-436 and MDA-MB-231 cancer cells. Thymoquinone significantly upregulated the expression of miR-603 in breast cancer cells. Studies have shown that miR-603 directly targets eEF-2K in cancer cells. The inhibition of NF-κB significantly enhanced miR-603 expression. Systemically administered thymoquinone effectively inhibited tumor growth in experimental mice implanted orthotopically with MDA-MB-231 and MDA-MB-436 cancer cells. In particular, levels of eEF-2K were found to be reduced in the tumor tissues of xenografted mice [56]. 

Thymoquinone and doxorubicin worked effectively and stimulated the levels of miR-375 and miR-16 in HepG2 and Huh7 cancer cells. Both miR-375 and miR-16 induced an increase in caspase-3 and a simultaneous reduction in the levels of BCL-2 [100]. 

Thymoquinone-loaded, hyaluronic-acid-conjugated Pluronic^®^ P123- and F127-co-polymer nanoparticles have been shown to be efficient against triple-negative breast cancer cells [101]. Thymoquinone nanoparticles induced disruption of the stress fibers by downregulating Rho and Rac1 in MDA-MB-231 cancer cells. These nanoparticles stimulated the expression of the tumor suppressor miR-361 in MDA-MB-231 cancer cells. Importantly, miR-361 directly targeted Rac1 and Rho. Thymoquinone-loaded nanoparticles markedly suppressed the number of pulmonary metastatic nodules in mice orthotopically injected with 4T1 cancer cells [101].

Transferrin-decorated thymoquinone-loaded PEG-PLGA nanoparticles triggered the upregulation of miRNA-16 and miRNA-34a via p53 in H1299 cancer cells [82]. Thymoquinone-loaded nanoparticles reduced the migration of p53 (wild-type) expressing lung cancer cells. These nanoparticles induced shrinkage of the tumor mass in mice injected with A549 cancer cells [102]. 

Likewise, PEGylated thymoquinone nanoparticles have also been demonstrated to stimulate the expression of miR-34a through p53 in cancer cells [103]. miR-34a directly targeted Rac1, followed by actin depolymerization. Thereafter, the depolymerization of actin further disrupted the actin cytoskeleton, which significantly reduced filopodia and lamellipodia formation on cell surfaces, thus retarding the migration of cancer cells. PEGylated thymoquinone nanoparticles significantly suppressed tumor weight and tumor volume in tumor-bearing mice. These nanoparticles efficiently enhanced the levels of catalase and superoxide dismutase (SOD) in tumor-bearing mice. PEGylated thymoquinone nanoparticles prevented oxidative stress, which further ameliorated the overall systemic toxicities [103]. 

## 12. Metastasis Inhibitory Role of Thymoquinone: Animal Model Studies

TQFL12, a new derivative, is more effective compared to thymoquinone. TQFL12 caused shrinkage of tumor xenografts in mice orthotopically implanted with 4T1 cancer cells [104]. 

Tristetraprolin (TTP) is a well-known AU-rich element-binding protein. TTP has been demonstrated to exert tumor-suppressive effects [105]. MUC4 was destabilized by direct binding of tristetraprolin to ARE in 3’UTR of MUC4 mRNA. Thymoquinone treatment reduced colony formation. However, the colony-forming ability of TTP-silenced cancer cells was found to be considerably enhanced. Thymoquinone treatment or TTP overexpression significantly reduced cancer metastasis and combinatorial treatment synergistically reduced pulmonary metastatic nodules [105]. 

Indirubin-3-monoxime and thymoquinone severely hampered tumor growth in subcutaneous A549 xenograft models. Levels of p-AKT and p-mTOR were reduced in the tumor tissues. Both individual and combinatorial treatments caused an increase in caspase-3 and p53 levels in the tumor tissues [106]. 

Thymoquinone suppressed VEGF-mediated activation of ERK. However, thymoquinone did not inactivate VEGFR2. Thymoquinone abolished tumor growth in mice subcutaneously transplanted with PC3 cancer cells. Thymoquinone significantly inhibited tumor angiogenesis as it effectively reduced the number of blood vessels in the tumor mass [107]. 

The constitutive activation of NLRP3 results in auto-inflammation characterized by sustained systemic and local inflammation mediated by interleukin-1β [108]. Glyburide is an inhibitor of NLRP3 inflammasomes. Glyburide blocks NLRP3 activation and secretion of IL-18 and IL-1β. Thymoquinone significantly decreased the expression of NLRP3 inflammasomes in B16F10 and A375 melanoma cells. Inflammasomes elicited the proteolytic maturation and secretion of IL-1β and IL-18 through caspase-1. Thymoquinone decreased the proteolytic cleavage of pro-caspase-1 in B16F10 and A375 melanoma cells. LPS/ATP are NLRP3 inflammasome activators. LPS/ATP triggered the activation of NLRP3 inflammasomes, including caspase-1 activities, which enhanced the secretion of IL-18 and IL-1β, consequently resulting in an enhanced migratory ability of melanoma cells. There was a notable reduction in macroscopic metastatic nodules on the surface of the lungs in C57BL/6 mice injected with B16F10 melanoma cells [108]. 

## 13. Concluding Remarks

Increasingly, it is being acclaimed that the strategy of conducting both forward translation (the implementation of scientific discoveries into clinical practice) as well as reverse translation (the process of elucidation of the underlying mechanisms of clinical observations) significantly reinforces the capability of interdisciplinary researchers to design and develop highly efficient anticancer treatment strategies. Recent advancements in genomic technologies and rapidly evolving large gene expression datasets have enabled researchers to sharply resolve the gene signatures that characterize malignant phenotypes. On the translational forefronts, pioneering studies over the past two decades have spurred the acclamation of thymoquinone as an effective cancer chemopreventive agent. Certain hints have emerged which indicate thymoquinone-mediated inactivation of the SHH/GLI and TGFβ/SMAD pathways [109,110]. Furthermore, thymoquinone worked effectively in combination with vitamin D3 and 5-fluorouracil and increased the expression of TGFβ and SMAD4 in chemical-induced cancer models [111,112]. Additionally, it will be exciting to explore the regulation of long non-coding RNAs and circular RNAs by thymoquinone. These aspects will add and enrich various aspects of molecular oncology. However, the prolonged administration of thymoquinone has an oncogenic role in mice xenografted with ovarian cancer cells. Therefore, these aspects have to be kept in consideration, mainly in the context of ovarian cancer. Collectively, the thymoquinone-mediated regulation of oncogenic pathways is encouraging and advocates its pharmacological significance. Thymoquinone has been unraveled to a greater extent and future studies must converge on rationally designed clinical trials for a better analysis of thymoquinone in cancer chemoprevention. 

## Data Availability

Not applicable.
